# Association of intestinal mucosal barrier function with intestinal microbiota in Spleen-Kidney Yang Deficiency IBS-D mice

**DOI:** 10.3389/fmicb.2025.1567971

**Published:** 2025-04-29

**Authors:** Liwen Li, Qi Long, Na Deng, Zhoujin Tan

**Affiliations:** 1 School of Traditional Chinese Medicine, Hunan University of Chinese Medicine, Changsha, China; 2 Laboratory of Chinese Medicine Prescription and Syndromes Translational Medicine, Changsha, China

**Keywords:** Spleen-Kidney Yang Deficiency, IBS-D, intestinal mucosal barrier, intestinal mucosal microbiota, *Folium senna*

## Abstract

**Background:**

To establish and evaluate an IBS-D mouse model with Spleen-Kidney Yang Deficiency, explore the microecological mechanisms of IBS-D, and provide experimental evidence for the clinical diagnosis and treatment of IBS-D with Spleen-Kidney Yang Deficiency.

**Methods:**

SPF-grade female Kunming mice were used to establish an IBS-D model with Spleen-Kidney Yang Deficiency through *Folium senna-*adenine administration combined with restraint-clamping tail. (1) Clinical symptoms and signs were assessed using diagnostic criteria. (2) The small intestine structure was examined via Alcian blue staining, and intestinal barrier markers like D-LA (D-lactate) and DAO (diamine oxidase) were measured by ELISA to assess pathophysiological changes. (3) 16S rRNA gene sequencing was performed to analyze the intestinal microbiota.

**Results:**

(I) The model mice exhibited symptoms of IBS-D with Spleen-Kidney Yang Deficiency. (II) ELISA and alcian blue staining revealed elevated levels of D-LA and DAO activity in the model group, indicating damage to the intestinal mucosal barrier structure. (III) Analysis of the intestinal mucosal microbiota in the model group revealed differences in dominant and characteristic bacteria at various taxonomic levels compared with those in the normal group, reflecting an imbalance in the intestinal mucosal microbiota. (IV) *Lactobacillus* and *Lentilactobacillus* are associated with mucosal barrier damage in mice modeled by *Folium senna-*adenine administration combined with restraint-clamping tail.

**Conclusion:**

The combination of *Folium senna-*adenine administration with restraint-clamping tail can be used to successfully establish an IBS-D mouse model with Spleen-Kidney Yang Deficiency. This model leads to damage to the intestinal mucosal structure. *Streptococcus*, *Serratia*, *Helicobacter*, *Phocaeicola*, and *Desulfomicrobium* may serve as potential biological markers for the intestinal mucosal microbiota.

## 1 Introduction

Irritable bowel syndrome (IBS) is one of the most common functional gastrointestinal disorders (FGIDs) in clinical practice (Chang and Talley, 2010). According to the latest Rome IV diagnostic criteria (Hellström and Benno, 2019), IBS is defined as recurrent abdominal pain occurring at least once a week for a minimum of 6 months, with at least two of the following criteria met: (1) abdominal pain related to bowel movements; (2) abdominal pain associated with a change in stool frequency; and (3) abdominal pain accompanied by a change in stool form or appearance. IBS can be further classified into four subtypes on the basis of the primary abnormal bowel habits of patients (Han, 2019): diarrhea-predominant (IBS-D), constipation-predominant (IBS-C), mixed (IBS-M), and unclassified (IBS-U). Currently, the etiology and pathogenesis of IBS remain unclear, with common contributing factors (Chen, 2019), including visceral hypersensitivity, intestinal infections, psychological factors, dietary habits, dysbiosis of the intestinal microbiota, and genetic factors. IBS-D is a prevalent subtype of IBS, accounting for approximately 23.4–40% of all IBS cases. The consensus opinion of traditional Chinese medicine (TCM) experts on IBS in 2017 categorized IBS-D into five patterns: liver qi stagnation with spleen deficiency, spleen deficiency with dampness, Spleen-Kidney Yang Deficiency, spleen and stomach damp heat, and mixed cold and heat (Bian et al., 2024). Spleen-Kidney Yang Deficiency is one of the main patterns in TCM for IBS-D. Spleen-Kidney Yang Deficiency is characterized by chronic diarrhea, loose or watery stools, abdominal coldness and pain, poor appetite, fatigue, lumbar soreness, and intolerance to cold. Additionally, patients may present with pale complexion, cold extremities, and deep, thready pulse, reflecting impaired yang energy and metabolic dysfunction. These pathological features contribute to the recurrent and refractory nature of diarrhea associated with Spleen-Kidney Yang Deficiency. The mechanism of this condition is not yet fully understood; however, current research suggests that it may be closely related to dysbiosis of the intestinal microbiota. Changes in the quantity and ratio of pathogenic bacteria to beneficial bacteria in the intestine can affect the stability of the intestinal microbiota, thereby influencing intestinal function (Ji et al., 2022; Ding and Sun, 2019).

The intestinal microbiota is a vast and diverse community of microorganisms that reside in the human intestine and is primarily composed of Firmicutes, Bacteroidetes, Proteobacteria, and Actinobacteria, among others. Among these, Firmicutes and Bacteroidetes are the most abundant, accounting for more than 90% of the total microbial population, and are crucial for maintaining gastrointestinal homeostasis (Shrestha et al., 2022). Under normal circumstances, the intestinal microbiota exists in a balanced state of mutual restraint and interdependence within the intestine, thereby stabilizing the intestinal microecosystem (Chong et al., 2019). Research has shown that dysbiosis of the intestinal microbiota is commonly observed in patients with diarrhea-predominant irritable bowel syndrome (IBS-D), characterized by an increase in Enterobacteriaceae and a decrease in *Lactobacillus*, *Bifidobacterium*, and *Prevotella* species (Tao, 2023), along with significant reductions in microbial density and diversity (Ji et al., 2016). The intestinal mucosa serves as the largest contact surface between the internal environment of the animal body and the substances within the intestinal lumen. It consists of mucosal epithelial cells, tight junctions between cells, and a biofilm, forming a robust mucosal immune system. In addition to facilitating nutrient absorption, it effectively prevents the invasion of pathogenic bacteria and food antigens in the intestine, thus protecting the health of the organism (Long et al., 2020). The mechanisms underlying intestinal microbiota dysbiosis in diarrhea-predominant irritable bowel syndrome (IBS-D) remain unclear, particularly the specific microecological regulatory networks associated with Spleen-Kidney Yang Deficiency Syndrome, a core pathogenesis in traditional Chinese medicine (TCM). Current studies indicate (In et al., 2016; O'Keefe, 2008) that most IBS patients experience damage to the intestinal mucosal barrier and increased mucosal permeability. Dysbiosis of the intestinal microbiota can impact the intestinal mucosal barrier by altering the function of intestinal epithelial cells, the permeability of the intestinal mucosa, and the expression of related proteins (Zhou J. J. et al., 2024), yet the dynamic evolution of Spleen-Kidney Yang Deficiency Syndrome-related microbiota, host-microbe co-metabolic mechanisms (e.g., short-chain fatty acid metabolism), and the microbiological basis of “Yang Deficiency-induced diarrhea” remain unelucidated. Existing animal models fail to simulate TCM syndrome progression patterns, resulting in weak associations between microbiota findings and biological targets of Yang-warming and spleen-invigorating therapies. This study establishes a novel Spleen-Kidney Yang Deficiency Syndrome-IBS-D mouse model integrating disease and syndrome features, aiming to reveal Spleen-Kidney Yang Deficiency Syndrome-specific microecological mechanisms and provide microbiological evidence for the TCM therapeutic principle of “reinforcing fire (Yang) to nourish earth (Spleen).”

Adenine is converted into poorly soluble 2,8-dihydroxyadenine under the action of xanthine oxidase (Zhu et al., 2022). After deposition in the renal tubules, it causes obstruction, inhibiting the excretion of nitrogenous compounds from the body (Klinkhammer et al., 2020). This leads to mechanical damage to the glomeruli and tubular functions, resulting in renal failure and affecting the body’s energy metabolism processes (Li et al., 2022b). Consequently, pathological states such as cold intolerance, a preference for warmth, lethargy, and weight loss may occur (Li et al., 2022a). *Folium senna* is commonly used in traditional Chinese medicine as a bitter and cold laxative. The main laxative components are sennosides A and B, which can cause increased intestinal motility, leading to diarrhea (Guan et al., 2021). Our research team reported that mice subjected to *Folium senna*-induced models presented significant diarrhea symptoms, and the model group presented dysbiosis of the intestinal mucosal microbiota (Zhang et al., 2020). Studies have shown that *Folium senna-*adenine administration combined with the restraint-clamping tail method increases intestinal sensitivity in rats, resulting in intestinal dysfunction and emotional disturbances (Liu et al., 2020). This method is suitable for producing irritable bowel syndrome models, with a short and straightforward modeling time (Tang et al., 2012). Furthermore, a comparison of the effects of different doses and durations of adenine and *Folium senna* on renal and intestinal function in mice revealed that adenine (75 mg/(kg•d), administered orally for 14 days) combined with *Folium senna* (10 g/(kg•d), administered orally for 7 days) significantly damaged the renal structure and intestinal function of the mice (Li et al., 2023; Deng et al., 2024). In this study, a model of Spleen-Kidney Yang Deficiency syndrome with diarrhea was established via *Folium senna-*adenine administration combined with restraint-clamping tail. By exploring the characteristics of the intestinal mucosal microbiota and the correlation between specific bacteria and the mucosal barrier, this research aims to provide scientific evidence for the treatment of diarrhea associated with the Spleen-Kidney Yang Deficiency syndrome from the perspective of the intestinal mucosal microbiota.

## 2 Materials and methods

### 2.1 Experimental animals

Twenty-two 4-week-old female Kunming mice (weighing 18–22 g) were purchased from Hunan Slackes Experimental Co., Ltd. (License No. SCXK [Xiang] 2019-0004). All the mice were kept in the Animal Experiment Center of Hunan University of Chinese Medicine (room temperature, 23–25°C; relative humidity, 50–70%; light/dark cycle, 12 h), with free access to food and water. The breeding feed for the experimental mice was provided by the Experimental Animal Center of Hunan University of Chinese Medicine and produced by Beijing Huafukang Biotechnology Co., Ltd.; the main components included crude protein ≥20%, crude fat ≥4%, crude fiber ≤5%, crude ash ≤8%, moisture ≤10%, lysine ≥1.3%, calcium 0.6–1.8%, phosphorus 0.6–1.2%, and sodium chloride 0.3–0.8%, ensuring cleanliness and contamination-free status. The feed was certified under the Beijing Feed Certificate: (2019) 06076. All animal experiments were conducted in accordance with the guidelines approved by the Animal Management and Use Committee of Hunan University of Chinese Medicine (License No. SYXK [Xiang] 2019-0009). The animal experiments were approved by the Animal Ethics and Welfare Committee of Hunan University of Chinese Medicine (HNUCM21-2404-26). To eliminate the influence of sex on the intestinal microbiota, only female mice were selected for this study (Wu et al., 2022).

### 2.2 Experimental drugs and their preparation

(1) Adenine (Changsha Yarsun Biotechnology Co., Ltd., Batch No. EZ7890C450). Preparation of adenine suspension (Xiao et al., 2008): Adenine was prepared using sterile water at a concentration of 5.625 mg/mL, proportional to the suspension concentration, and was freshly prepared daily for use.

(2) *Folium senna* (Anhui Shenghaitang Traditional Chinese Medicine Co., Ltd., Batch No. 2019060561). Preparation of *Folium senna* decoction (Xie et al., 2022): A suitable amount of *Folium senna* was placed in a cooking vessel, and enough water was added to cover the leaves, allowing them to soak for 30 min. Five times the volume of water was subsequently added to the container, which was subsequently boiled for 30 min. The filtered residue was then combined with an appropriate amount of water and further boiled for 15 min. The two decoctions were mixed and concentrated to achieve a final concentration of 1 g/mL decoction, which was stored in a refrigerator at 4°C.

### 2.3 Reagents

A D-LA (D-lactate) ELISA Kit (Jiangsu Jingmei Biotechnology Co., Ltd., No. JM-11669M1) and a DAO (diamine oxidase) ELISA Kit (Jiangsu Jingmei Biotechnology Co., Ltd., No. JM-02511M1) were used.

### 2.4 Animal grouping and model preparation

Following a 3-day acclimatization period, 22 female mice were randomly allocated into a normal group (CC, *n* = 11) and a model group (CM, *n* = 11). Starting from day 1 of modeling, the mice in model group received daily oral gavage of ice adenine suspension (50 mg/kg/day) for 14 consecutive days, supplemented with *Folium senna* decoction (10 g/kg/day) from day 10 to 14 (once daily) (Li et al., 2022a). The mice in normal group were administered 0.4 mL of sterile water via daily gavage for 14 days. From modeling day 8, restraint-clamping stress was applied to model group mice daily at 9 AM as follows: Limbs were immobilized using 50 mL centrifuge tubes, while a hemostatic clamp was applied to 1/3 of the tail. This combined stress protocol lasted 1 h/day for 7 consecutive days (Liu et al., 2024).

### 2.5 Model evaluation criteria

The IBS-D model, characterized by Spleen-Kidney Yang Deficiency, can be evaluated based on general behavioral observations, histopathological analysis, and open field test (OFT) outcomes.

(1) Referring to the “Standards for Differentiating Deficiency Syndromes in Traditional Chinese Medicine” (Shen, 1983) and the “Expert Consensus on the Diagnosis and Treatment of Diarrhea in Traditional Chinese Medicine (2017)” (Bian et al., 2024), the evaluation of kidney Yang deficiency and cold hypersensitivity was based on behaviors such as clustering, cold limbs, and anal temperature. Irritability and aggressive behavior are assessed to evaluate stress-induced irritability.

(2) Open field test: On the 14th day of modeling, the mice were placed in the testing room for 30 min of acclimatization. Using a random number table, 5 mice from each group were selected for detection in the KSYY-OP-V4.0 mouse open field real-time detection and analysis system. The movement distance and average speed of the mice were observed over a 5-min period. After each trial, the feces and urine were removed, and the testing chamber was wiped with 75% ethanol. The chamber was allowed to air out before this process was repeated 5 times, and the average values were recorded (Qiao et al., 2023).

### 2.6 General behavioral observations

During the experiment, the following parameters of the mice were observed: body weight, food intake, water consumption, fecal characteristics, anal temperature, mental state, activity levels, and changes in physical signs.

### 2.7 Pathological sections of the small intestine

Small intestine tissue was immersed in 4% paraformaldehyde for fixation, followed by the preparation of paraffin sections. The paraffin sections were deparaffinized with xylene, hydrated through an alcohol gradient, and then treated with an acetic acid solution applied to the tissue block for 3 min at room temperature. Next, the sections were incubated in Alcian blue staining solution for 20 min. After rinsing with distilled water, a nuclear red staining solution was added. Upon confirming successful staining, dehydration and clearing were performed, followed by mounting with neutral gum. Images were acquired via a microscope.

### 2.8 D-LA and DAO ELISA analysis

Whole blood samples (0.7–1.5 mL) were collected from each cage (*n* = 5 mice per group) via standard serum collection tubes (red-capped vacuum blood collection tubes). After centrifugation at 3,000 r/min for 10 min, the serum was separated and transferred to sterilized centrifuge tubes. The blood samples designated for ELISA were allowed to stand at room temperature for 30 min. The ELISA procedure was conducted according to the instructions provided in the kit, including the setup of the plate layout, sample addition, enzyme addition, incubation, washing, color development, reaction termination, and machine detection.

### 2.9 16S rRNA high-throughput sequencing

After successful modeling, five mice were selected from each group. Under sterile conditions, the abdominal cavity of each mouse was opened to extract the small intestine. The small intestine was cut open along its long axis, rinsed with sterile saline, and dried with sterile filter paper, and the mucosa was scraped off using a sterilized coverslip. The samples were collected with a sterilized coverslip (Li et al., 2021a; He et al., 2019; Shen et al., 2025). Each sample was placed in a 1.5 mL sterilized centrifuge tube, labeled, weighed, and then stored at −80°C for intestinal mucosal microbiota analysis (Li C. et al., 2022). Total genomic DNA was extracted from the intestinal mucosal samples via a bacterial DNA kit (OMEGA, United States). The quantity and quality of the extracted DNA were assessed via agarose gel electrophoresis and a NanoDrop NC2000 spectrophotometer (Thermo Fisher Scientific, Waltham, MA, United States). The nearly full-length 16S rRNA gene of bacteria was amplified via PCR via the forward primer 338F (5′-ACTCCTACGGGAGGC AGCA-3′) and the reverse primer 806R (5′-GGACTACHV GGGTWTCTAAT-3′). The Q5 high-fidelity DNA polymerase (New England BioLabs, United States) was employed for the amplification of the 16S rRNA gene through polymerase chain reaction (Fang et al., 2025). The PCR products were verified via 2% agarose gel electrophoresis and purified via the Axygen ®AxyPrep DNA Gel Extraction Kit. The recovered PCR amplification products were quantified fluorometrically via the Quant-iT PicoGreen dsDNA Assay Kit. On the basis of the fluorescence quantification results, samples were mixed proportionally according to the sequencing requirements for each sample (Yuan et al., 2020). Sequencing was performed by Paiseno Biological Co., Ltd. (Shanghai, China).

### 2.10 Bioinformatics and statistical analysis

(1) Species notes: Using the DADA2 denoising pipeline within QIIME2 software, sequence denoising was performed, effectively equivalent to 100% similarity clustering, and generating ASV feature sequences and an ASV table with singletons removed. Taxonomic annotation of ASV feature sequences or representative OTU sequences was conducted using QIIME2’s classify-sklearn algorithm. The annotated taxonomic information was further analyzed to generate ASV/OTU Venn diagrams, alpha diversity, beta diversity, dominant microbial composition, characteristic microbial taxa, and functional predictions.

(2) Alpha diversity analysis: The Chao1 index, observed species index, Shannon index, and Simpson index of each group were calculated to compare the richness and evenness of ASVs between different samples.

(3) Beta diversity analysis: The Bray–Curtis distance was used to analyze the changes in microbial community structure between the samples. Principal coordinate analysis (PCoA) and non-metric multidimensional scaling (NMDS) were used for visualization.

(4) Characteristic microbiota analysis: QIIME2’s software was used to obtain the composition and abundance tables of each sample at different taxonomic levels, which were presented as bar charts. Linear discriminant analysis effect size (LEfSe) was used with default parameters to detect differential abundance among different taxa. The random forest analysis of samples from different groups was performed using QIIME2’s default settings.

(5) Correlation analysis: The Spearman correlation coefficient among characteristic bacteria was calculated. The correlation network was constructed by Cytoscape 3.7.2 software to explore the synergistic/competitive relationship among characteristic bacteria. Redundancy analysis (RDA) was used to investigate the interactions between the characteristic flora of intestinal contents and environmental factors (Zhou M. et al., 2024).

(6) Functional predictive analysis: PICRUSt2 was used to predict the functional abundance of samples in the KEGG (Kyoto Encyclopedia of Genes and Genomes) database and to perform LEfSe analysis to identify the metabolic pathways with different abundances between groups. Spearman analysis was used to explore the correlation between blood lipids and oxidative indices with the intestinal microbiota in the small intestine (Yang et al., 2024; Zhang et al., 2021).

### 2.11 Statistical analysis

Statistical analysis was performed via SPSS version 25.00. The data from each group are expressed as the means ± standard deviations. If the two groups of data followed a normal distribution and had equal variances, an independent samples *t*-test was conducted. If the data did not meet the assumptions of normality and homogeneity of variance, the Wilcoxon rank-sum test was used. A *p*-value of <0.05 or <0.01 was considered statistically significant; otherwise, the differences were deemed not statistically significant (Li et al., 2021b). In this experiment, the D-LA and DAO levels as well as the Alizarin Red staining results all followed a normal distribution, so independent sample t-tests were used for statistical analysis.

## 3 Results

### 3.1 Effects of IBS-D mice with Spleen-Kidney Yang Deficiency modeling on behavioral phenotypes

#### 3.1.1 Changes of symptoms and signs in IBS-D mice with Spleen-Kidney Yang Deficiency

**Figure 1:**
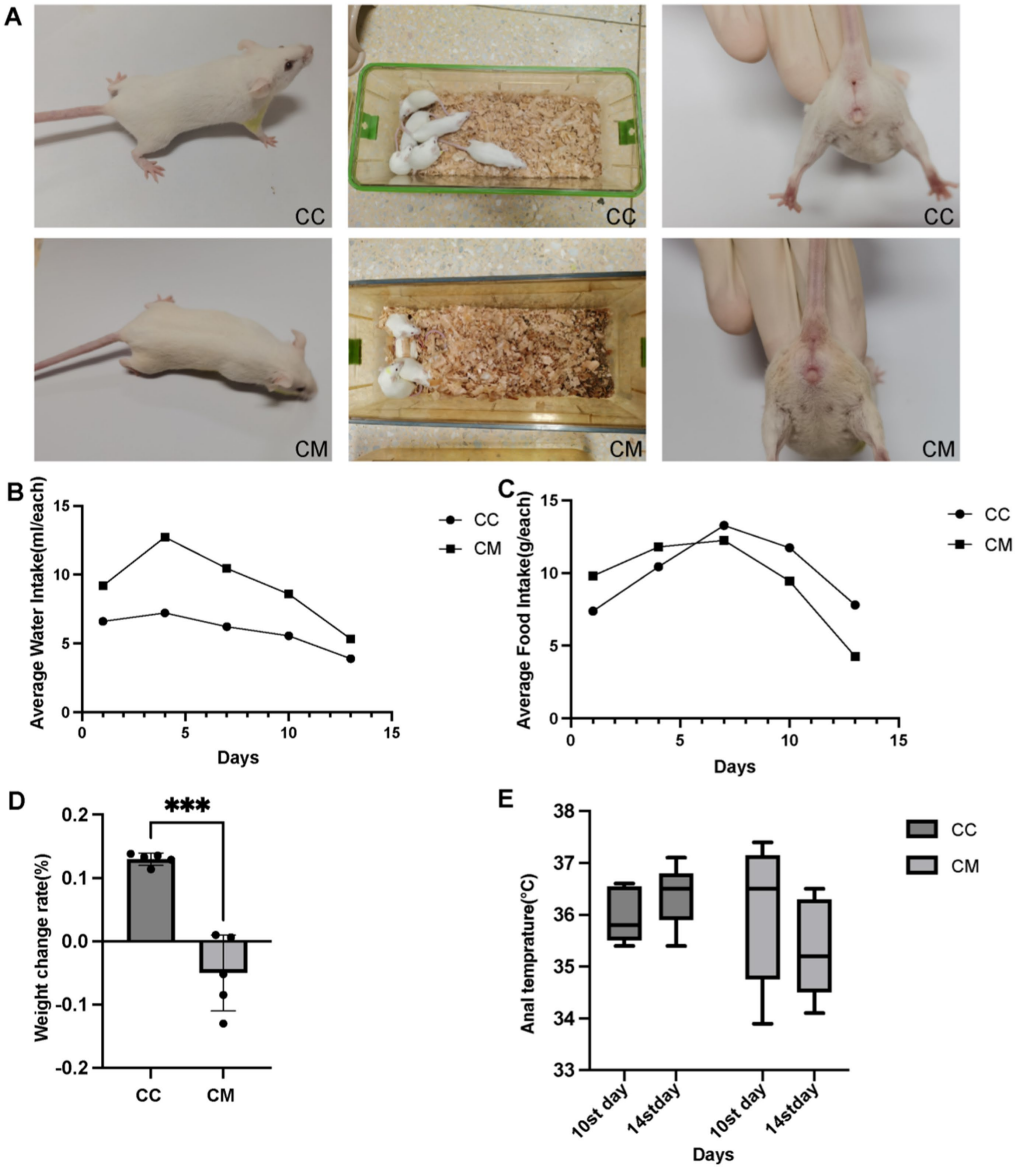
Spleen-Kidney Yang Deficiency IBS-D modeling induces alterations in Open Field Test performance in mice. **(A)** Changes in the symptoms and signs of the mice. **(B)** Water intake. **(C)** Food consumption. **(D)** Weight change rate. **(E)** Changes in anal temperature. CC: Normal group (*n* = 5); CM: Model group (*n* = 5). Weight change rate during the modeling period = (Weight at the end of modeling - Weight at the end of adaptation) / Weight at the end of adaptation × 100%. The values are expressed as the means ± standard deviations.

During the modeling period, normal group mice exhibited a normal mental state and voluntary activity, with responsive behaviors and smooth fur (Figure 1A), and an absence of huddling behavior. Their bedding remained dry, and fecal pellets were well-formed with normal moisture content and clean perianal regions. In contrast, model group mice exhibited a poor mental state, reduced voluntary activity, unkempt fur, hunched posture with persistent huddling behavior, increased bedding moisture, loose stools, and perianal fecal staining. These behavioral and physiological alterations demonstrate that the *Folium senna-*adenine administration combined with restraint-clamping tail modeling successfully induced behavioral dysfunction characteristic of intestinal barrier impairment.

#### 3.1.2 Changes of water intake and food consumption in IBS-D mice with Spleen-Kidney Yang Deficiency

On day 1 of modeling, there was little difference in water intake or food consumption between normal group and model group. Starting from day 1 of modeling (Figure 1B), the average water intake of model group was significantly greater than that of normal group. From day 7 of modeling (Figure 1C), the average daily food consumption of model group consistently remained lower than that of normal group.

#### 3.1.3 Determination of the rate of weight change in IBS-D mice with Spleen-Kidney Yang Deficiency

As shown in Figure 1D, the weight change rate of normal group mice was greater than that of model group mice (*p* < 0.001).

#### 3.1.4 Changes of anal temperatures in IBS-D mice with Spleen-Kidney Yang Deficiency

Compared with day 10 of modeling (Figure 1E), the rectal temperature of normal group mice increased on day 14 of modeling, whereas the rectal temperature of model group mice decreased on day 14 of modeling (*p* > 0.05).

#### 3.1.5 Open field test reveals behavioral alterations in IBS-D mice with Spleen-Kidney Yang Deficiency

**Figure 2:**
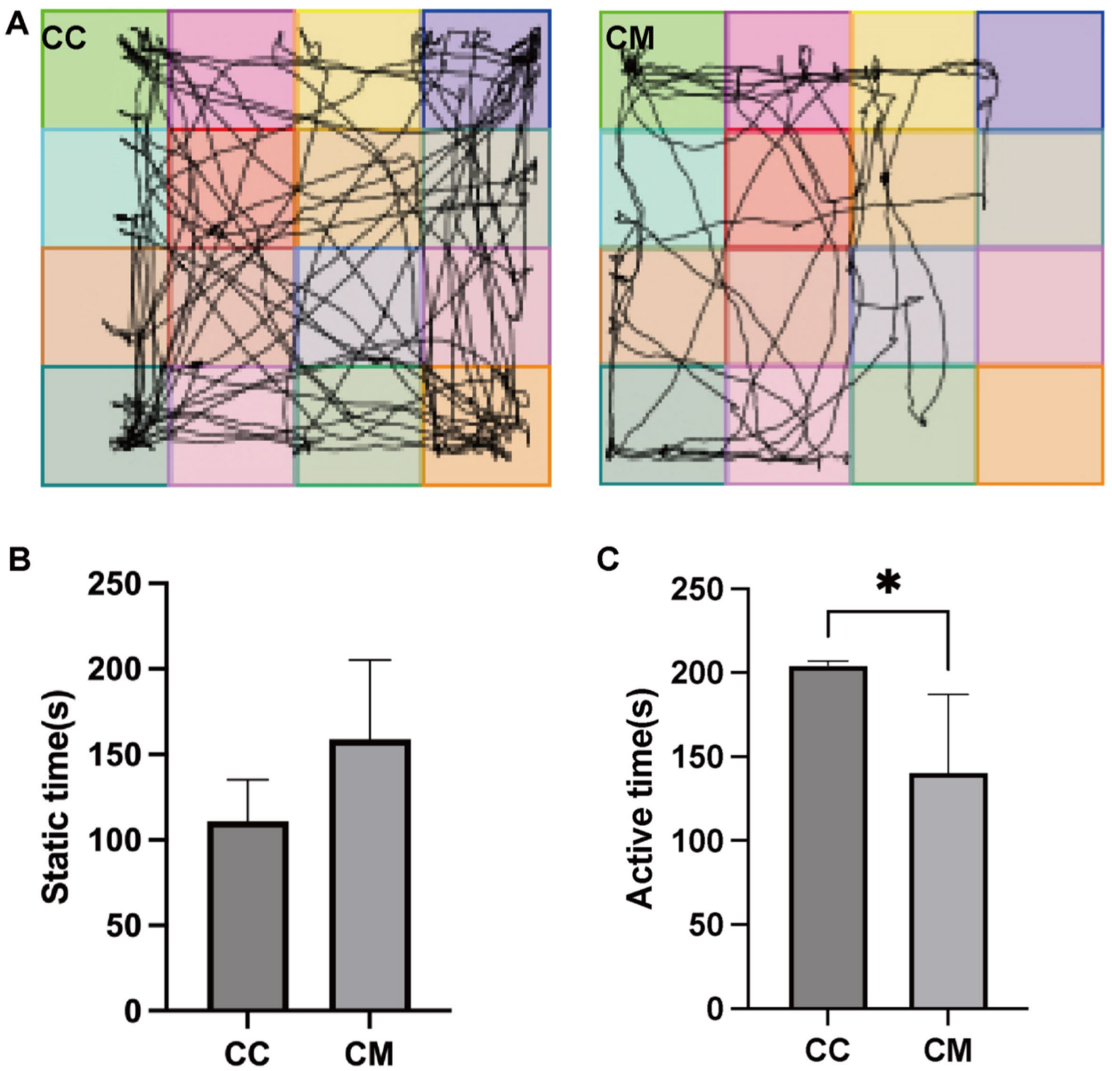
Spleen-Kidney Yang Deficiency IBS-D modeling induces behavioral alterations in mice. **(A)** Movement trajectories of mice in the open field test. **(B)** Static time of mice in the open field test. **(C)** Active time of mice in the open field test. CC: Normal group (*n* = 5); CM: Model group (*n* = 5). *p < 0.05.

As shown in Figure 2A, normal group of mice exhibited frequent activity, often passing through the central area, with their activity being primarily concentrated in the center and surrounding areas of the testing box. In contrast, model group mice showed sluggish responses, with their activity largely confined to the four corners of the testing box, and they did not form a closed movement trajectory even in the surrounding areas. The immobility time of model group mice was significantly greater than that of normal group mice (*p* > 0.05), whereas the activity time of normal group mice was significantly greater than that of model group mice (*p* < 0.05) (Figures 2B,C).

### 3.2 Effects of IBS-D mice with Spleen-Kidney Yang Deficiency modeling on the intestinal mucosal barrier

#### 3.2.1 IBS-D mice with Spleen-Kidney Yang Deficiency modeling causes small intestinal mucosal injury

**Figure 3:**
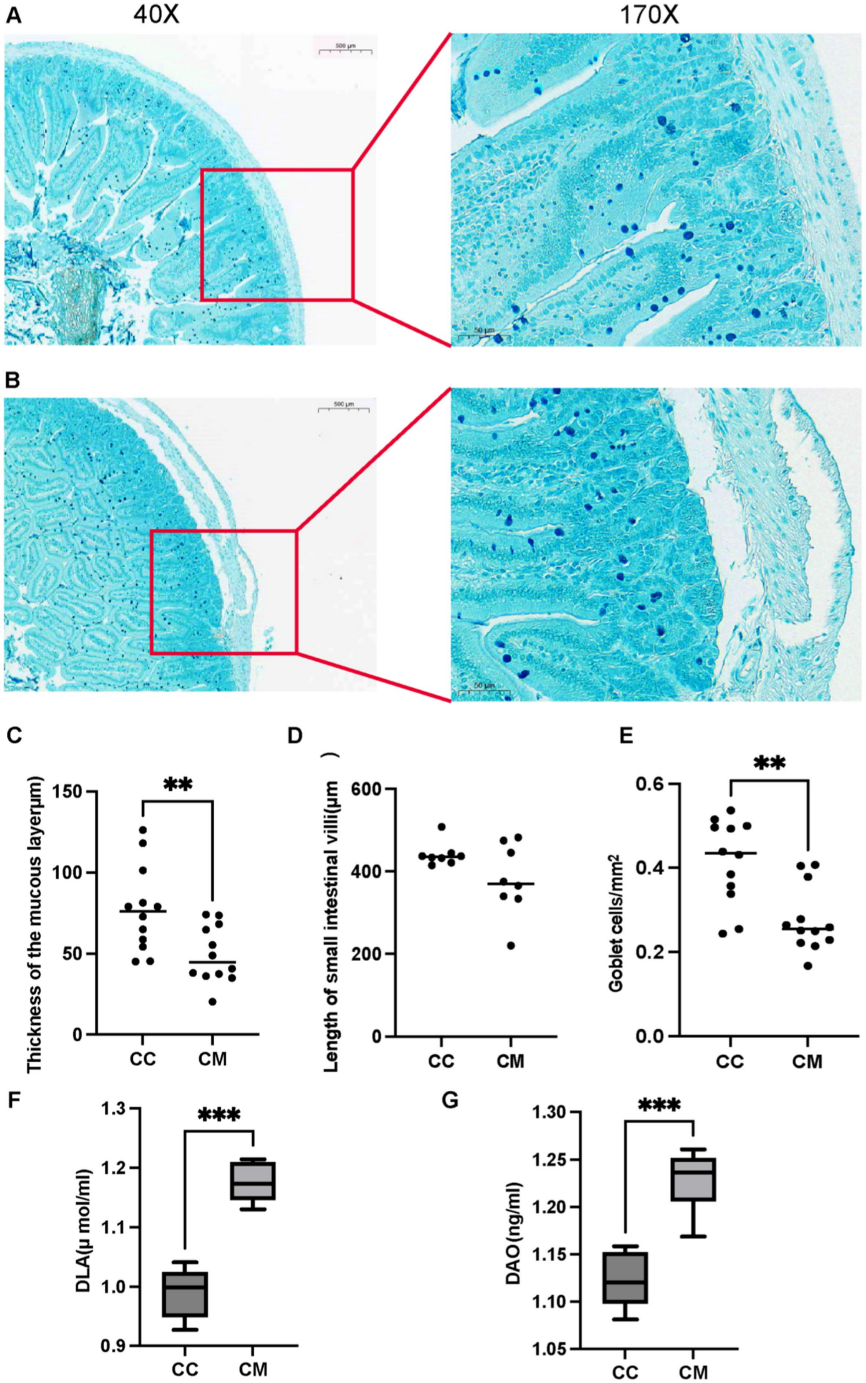
IBS-D mice with Spleen-Kidney Yang Deficiency modeling impairs intestinal mucosal barrier integrity. **(A)** Alcian blue staining of small intestinal sections from normal group. **(B)** Alcian blue staining of small intestinal sections from model group. **(C)** Thickness of the mucous layer. **(D)** Length of small intestinal villi. **(E)** Goblet cells. **(F)** Serum D-LA activity. **(G)** Serum DAO activity. CC: Normal group (*n* = 5); CM: Model group (*n* = 5). *p < 0.05, **p < 0.01, ***p < 0.001.

Mucin 2 (Muc2), the most abundant mucin constituting the mucus layer, is extensively glycosylated and secreted by goblet cells (Li D. et al., 2021), serving as the primary structural component of the intestinal mucus barrier (Zhang et al., 2024). The results of Alcian blue staining revealed well-defined crypt structures with abundant goblet cells in normal group. In contrast, model group exhibited pathological features including separation of the submucosal layer and lamina propria, muscular layer edema, disorganized or absent villous architecture, significant depletion of mucus-producing goblet cells, and marked thinning or complete absence of the mucus layer (Figures 3A,B). These results indicate that the Spleen-Kidney Yang Deficiency IBS-D modeling significantly reduced the thickness of the mucous layer (Figure 3C) (*p* < 0.05), the length of the small intestinal villi (Figure 3D) (*p* > 0.05), and the number of goblet cells (Figure 3E) (*p* < 0.001), collectively indicating compromised intestinal mucosal barrier integrity.

#### 3.2.2 IBS-D mice with Spleen-Kidney Yang Deficiency modeling aggravates intestinal mucosal barrier injury

Compared with those in normal group, the activities of D-LA and DAO in model group were significantly greater (*p* < 0.001) (Figures 3F–G). These findings indicate that the Spleen-Kidney Yang Deficiency syndrome IBS-D modeling affects intestinal mucosal barrier function in mice to some extent.

### 3.3 Quality assessment of mucosal microbiota sequencing data in IBS-D mice with Spleen-Kidney Yang Deficiency

**Figure 4:**
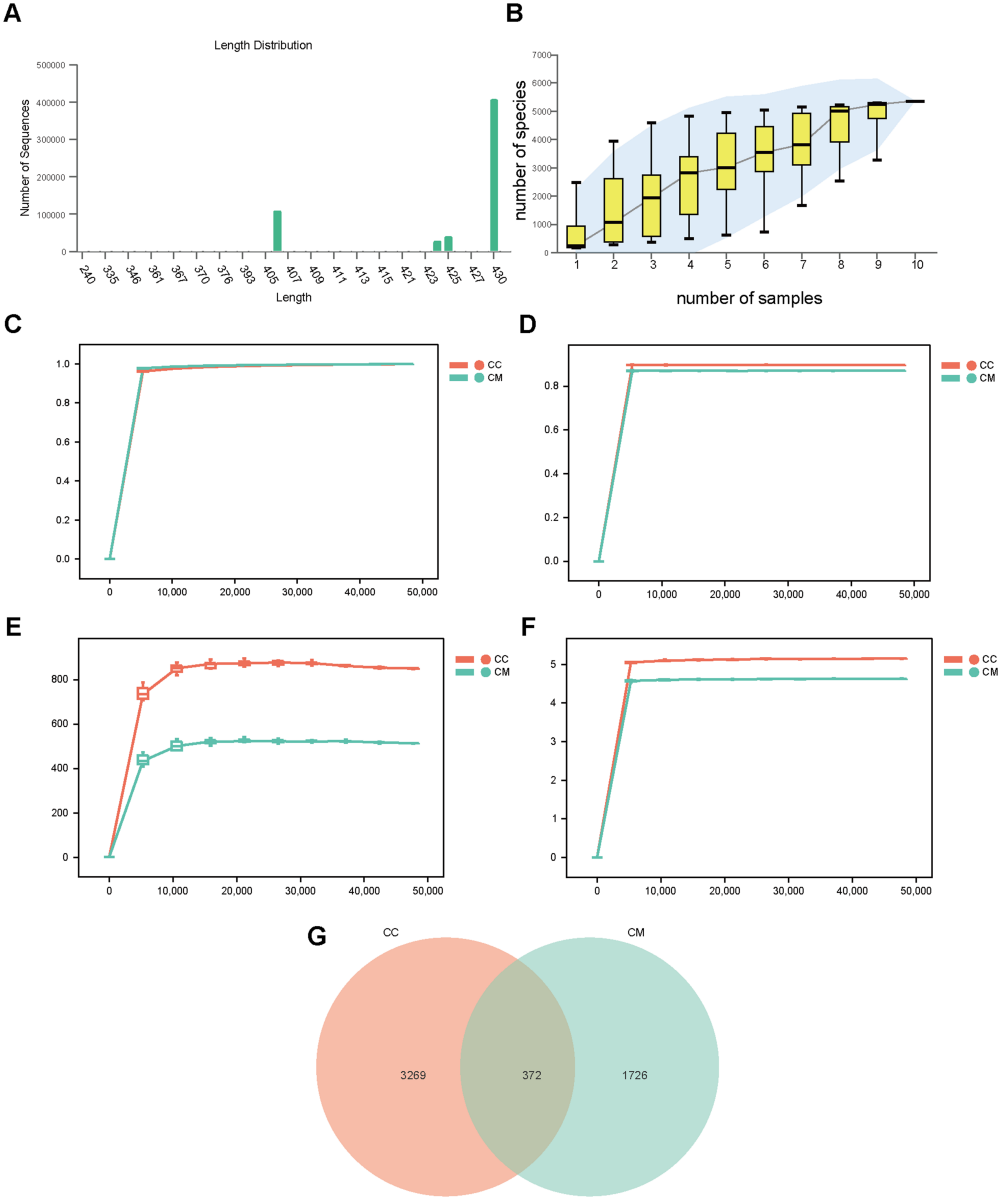
Quality assessment of sequencing data, OTU analysis, and Venn diagram visualization for intestinal mucosal microbiota characterization. **(A)** Sequence length distribution. **(B)** Species accumulation curve. **(C)** Observed species rarefaction curve. **(D)** Simpson rarefaction curve. **(E)** Chao1 rarefaction curve. **(F)** Shannon rarefaction curve. **(G)** Venn diagram. CC: Normal group (*n* = 5); CM: Model group (*n* = 5). The data are presented as the means ± standard deviations.

We analyzed the sequence length obtained from the mucosal microbiota sequencing of the mice, and the results revealed that the length distribution of the sequences across the samples was concentrated at approximately 403–430 bp (Figure 4A). To assess whether the samples represented the overall population, we conducted random sampling and plotted a species accumulation curve (Figure 4B). We found that as the sample size increased, the number of detected species significantly increased, and the curve was relatively steep. When the sample size reached a certain level, further increases did not lead to the detection of new species, and the curve began to flatten, indicating that the sample size in this experiment was sufficient to reflect the richness of the community.

We subsequently plotted rarefaction curves to analyze the sequencing depth of the experiment. As shown in Figures 4C–F, the rarefaction curves exhibited an inflection point, followed by a flattening trend as the sequencing depth increased, reaching a plateau phase. These findings suggest that the sequencing depth for both groups of samples was adequate, covering most biological species, and that the species richness of the tested samples was sufficient for subsequent research.

### 3.4 Effects of IBS-D mice with Spleen-Kidney Yang Deficiency on OTU richness in mouse intestinal mucosal microbiota

A Venn diagram was used to analyze the unique and shared OTUs between different sample groups, which visually depicted the similarity and uniqueness of the samples at the OTU level. Normal group shared 372 OTUs with model group. Normal group had 3,269 unique OTUs, whereas model group had 1726 unique OTUs. The total number of OTUs in normal group was 3,641, whereas the total number of OTUs in model group was 2,098 (Figure 4G), indicating that modeling increased the diversity and number of taxonomic units of the intestinal mucosal microbiota in the mice.

### 3.5 Effects of IBS-D mice with Spleen-Kidney Yang Deficiency on diversity and community structure of mouse intestinal mucosal microbiota

#### 3.5.1 Alpha diversity analysis

**Figure 5:**
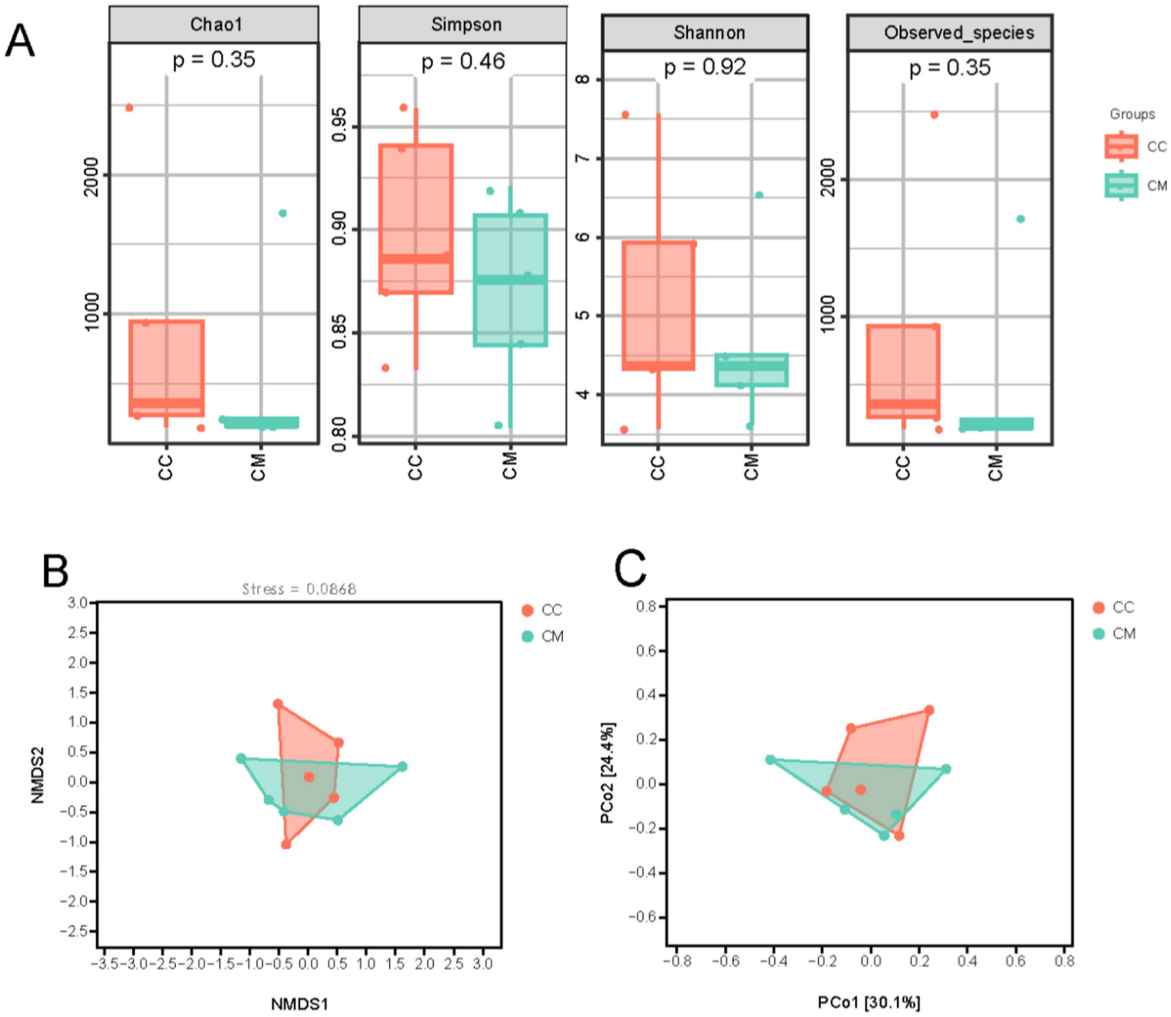
Effects of Spleen-Kidney Yang Deficiency IBS-D modeling on Alpha and Beta diversity of intestinal mucosal microbiota in mice. **(A)** Alpha diversity. **(B)** NMDS analysis. **(C)** PCoA. CC: Normal group (*n* = 5); CM: Model group (*n* = 5).

To comprehensively evaluate the alpha diversity of the microbial community, we selected the Chao1 and Observed_species indices to assess community richness. The Shannon and Simpson indices were used to evaluate community diversity (Figure 5A). Compared with normal group, model group presented greater Chao1, Simpson, Shannon, and observed species indices (*p* > 0.05). The results suggest that after modeling, there is a trend of change in the intestinal mucosal microbiome diversity of the mice in model group.

#### 3.5.2 Beta diversity analysis

Beta diversity reflects differences in species abundance and distribution and is used to explore the similarity in community distributions between different samples. Three main methods—NMDS and PCoA analysis based on an unweighted distance matrix—were employed for natural decomposition of the community data structure. As shown in the figures (Figures 5B,C), model group samples were separated from normal group samples, exhibiting distinct grouping and clustering phenomena. These findings indicate that the administration of *Folium senna-*adenine administration combined with restraint-clamping tail altered the homogeneity of the intestinal mucosal microbiota, indicating that the modeling process caused a shift in the overall structure of the intestinal mucosal microbiome.

### 3.6 Composition of dominant bacteria in the intestinal mucosa of IBS-D mice with Spleen-Kidney Yang Deficiency

**Figure 6:**
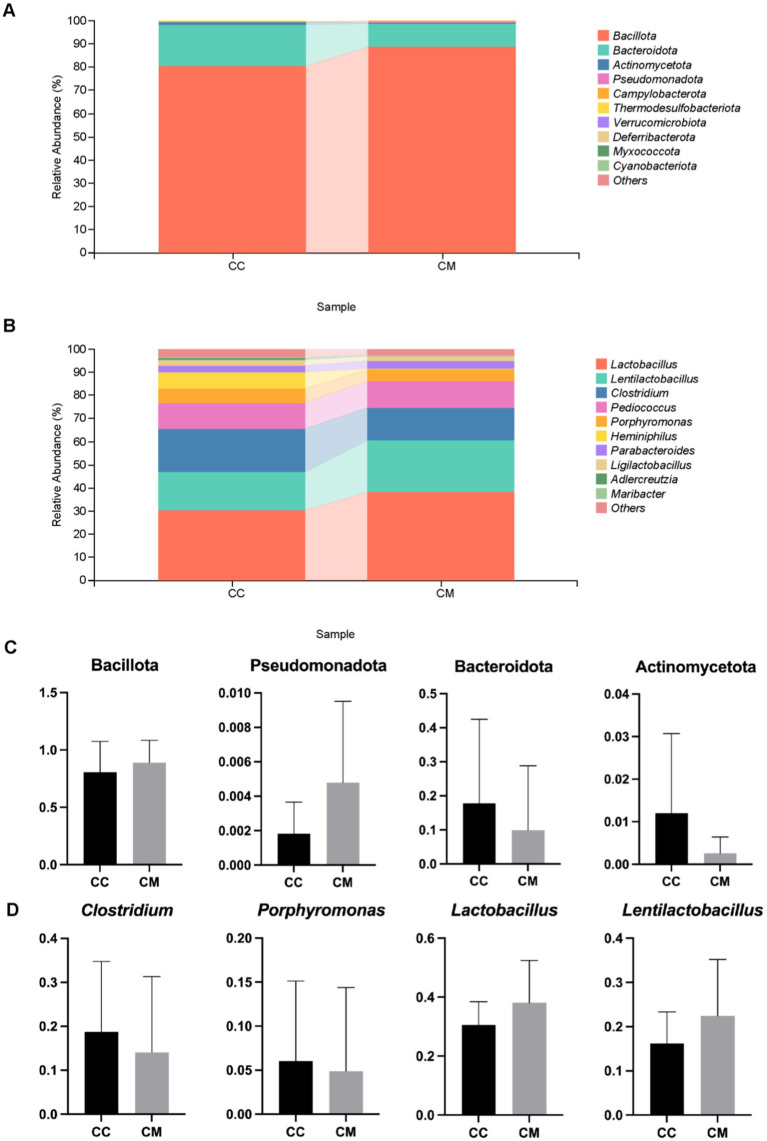
Effects of Spleen-Kidney Yang Deficiency IBS-D modeling on relative abundance of intestinal mucosal microbiota in mice. **(A)** Relative abundance of intestinal mucosal microbiota at the phylum level. **(B)** Relative abundance of intestinal mucosal microbiota at the genus level. **(C)** Dominant bacteria at the phylum level in the intestinal mucosa of the mice. **(D)** Dominant bacteria at the genus level in the intestinal mucosa of the mice. CC: Normal group (*n* = 5); CM: Model group (*n* = 5). The values are expressed as the means ± standard deviations.

We conducted a taxonomic analysis of the intestinal mucosal microbiota in the normal and model groups of mice and compared the relative abundance differences at the phylum and genus levels. Figure 6A shows that at the phylum level, the top five phyla in normal group were Bacillota, Bacteroidota, Actinomycetota, Thermodesulfobacteriota, and Pseudomonadota. In contrast, the top five phyla in model group were Bacillota, Bacteroidota, Pseudomonadota, Campylobacterota, and Actinomycetota. Compared with normal group, model group presented lower proportions of Bacteroidota and Actinomycetota, whereas the proportions of Bacillota and Pseudomonadota were greater, indicating a change in the composition of the dominant bacteria at the phylum level.

At the genus level (Figure 6B), the top five genera in normal group were *Lactobacillus*, *Clostridium*, *Lentilactobacillus*, *Pediococcus*, and *Heminiphilus*. In model group, the top five genera were *Lactobacillus*, *Lentilactobacillu*, *Clostridium*, *Pediococcus*, and *Porphyromonas*. Compared with those in normal group, the proportions of *Clostridium* and *Porphyromonas* were lower in model group, whereas the proportions of *Lactobacillus* and *Lentilactobacillu* were greater (Figure 6C). These findings indicate that the composition of the dominant bacteria at the genus level changed.

We further conducted a statistical analysis of the relative abundances of bacteria at the phylum and genus levels, with abundances greater than 1% in both normal group and model group (Figure 6D). Compared with those in normal group, the relative abundances of Bacillota and Pseudomonadota increased in model group, whereas those of Bacteroidota and Actinomycetota decreased. In model group, the abundances of *Clostridium* and *Porphyromonas* decreased, whereas those of *Lactobacillus* and *Lentilactobacillu* increased. In summary, the administration of *Folium senna-*adenine administration combined with restraint-clamping tail altered the composition of the dominant bacteria in the intestinal mucosa, with decreases observed in Bacteroidota, Actinomycetota, and *Clostridium* (*p* > 0.05).

### 3.7 Identification of signature microbial species in the intestinal mucosal microbiota of IBS-D mice with Spleen-Kidney Yang Deficiency

**Figure 7:**
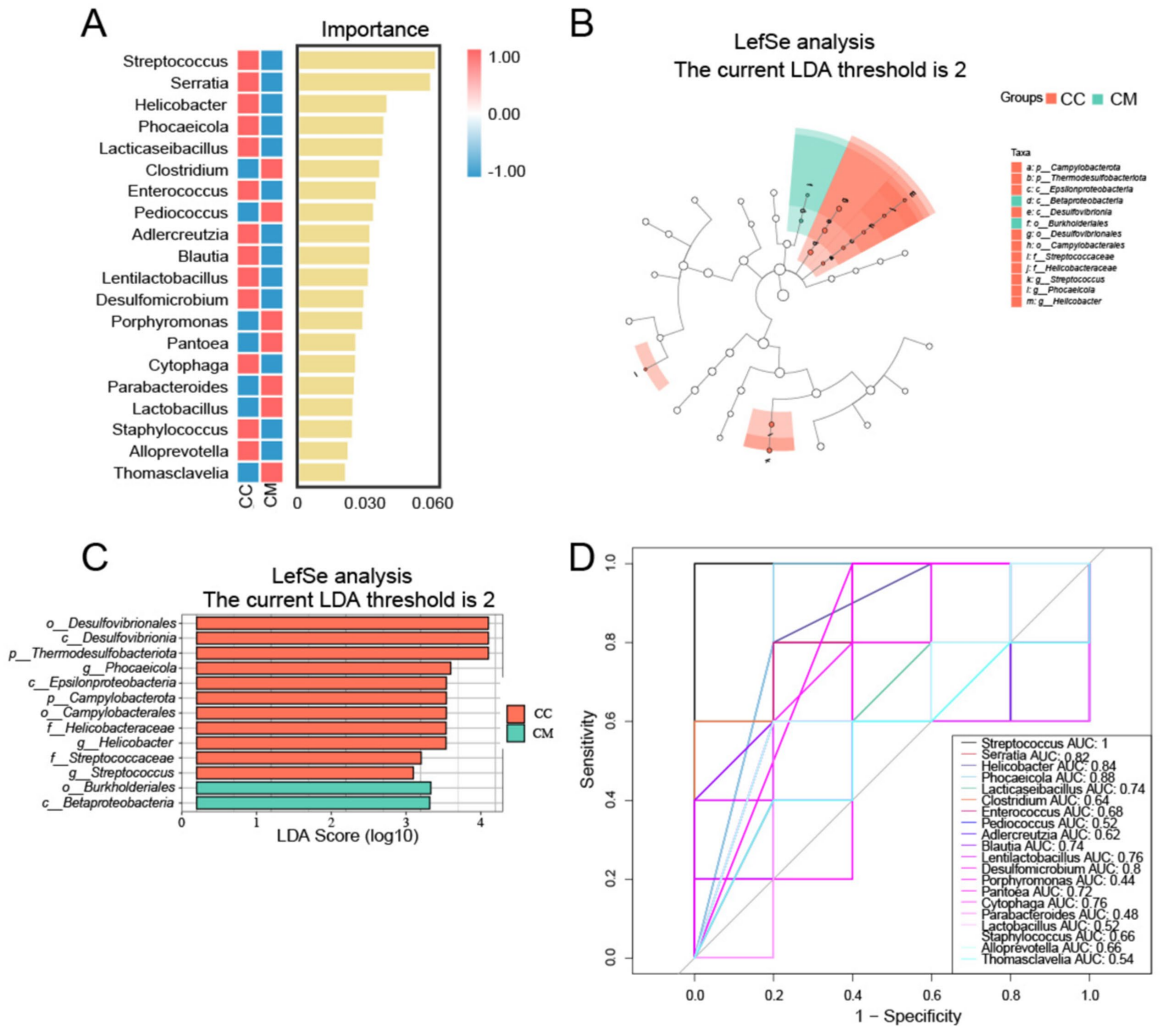
Analysis of characteristic bacteria in the intestinal mucosal microbiota. **(A)** Random forest plot at the genus level. **(B)** Bar chart. **(C)** LDA plot. **(D)** ROC curve for species. CC: Normal group (*n* = 5); CM: Model group (*n* = 5).

LEfSe analysis revealed differentially altered characteristic bacterial taxa, with LDA scores greater than 2. Characteristic bacteria were identified in both normal group and model group, with differences in abundance observed between the two groups. Normal group revealed 11 key differential factors, whereas model group revealed 2 key differential factors. At the genus level, Streptococcus, Helicobacter and Phocaeicola were identified as characteristic genera of normal group (Figures 7B,C). We subsequently constructed a random forest diagnostic model in which 20 bacterial genera were used to distinguish between normal group and model group (Figure 7A). ROC results indicated that Streptococcus, Serratia, Helicobacter, Phocaeicola, and Desulfomicrobium presented high AUC values (Figure 7D), suggesting that these genera may serve as potential biomarkers at the genus level of the intestinal mucosal microbiota for the diagnosis of IBS-D with Spleen-Kidney Yang Deficiency.

### 3.8 Functional alterations of intestinal mucosal microbiota in IBS-D mice with Spleen-Kidney Yang Deficiency

**Figure 8:**
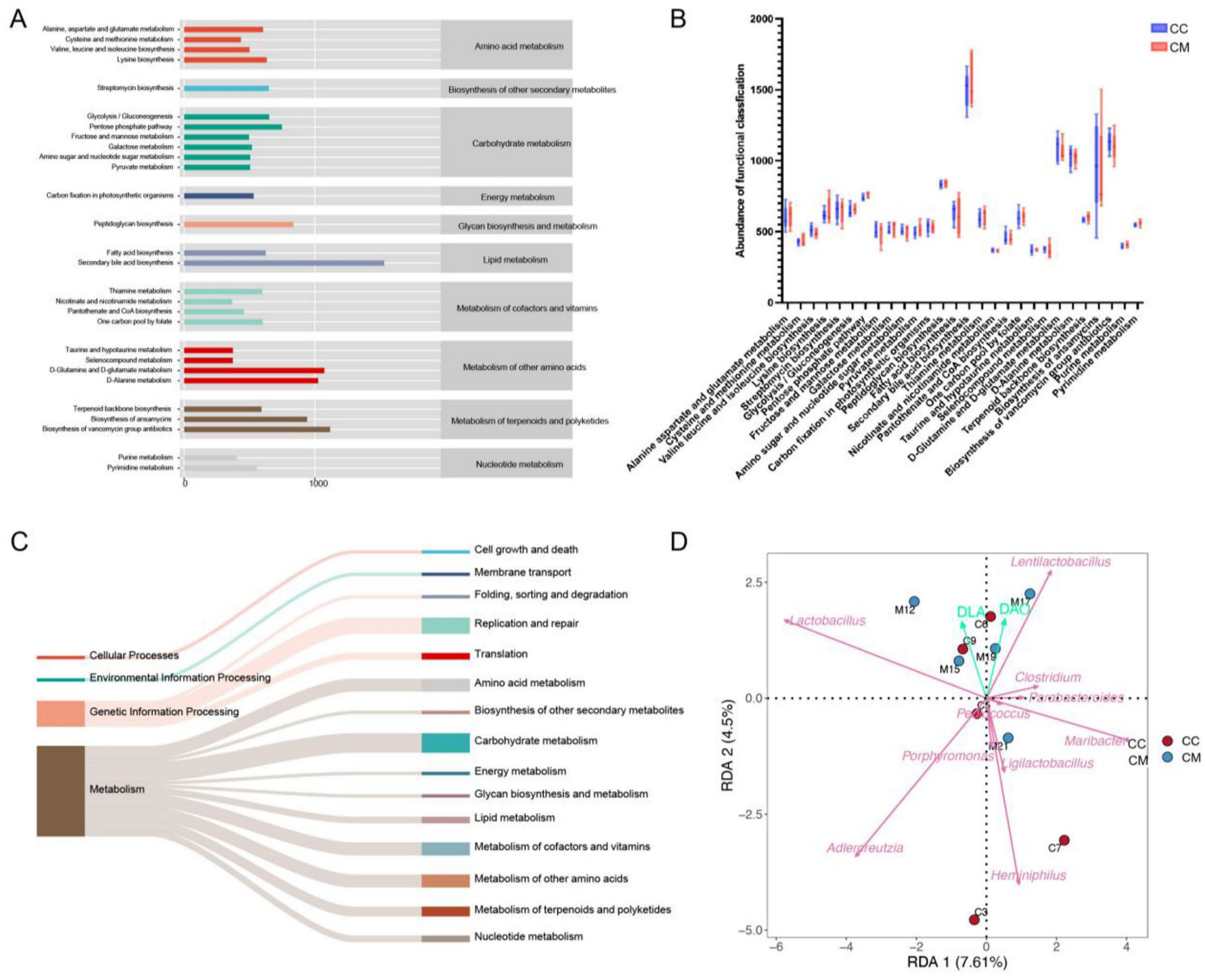
Functional analysis of the intestinal mucosal microbiota and RDA analysis. **(A)** Predicted abundance of KEGG functions. **(B)** Box plot. **(C)** Sankey diagram tracking. **(D)** RDA. CC: Normal group (*n* = 5); CM: Model group (*n* = 5).

To further investigate the role of the intestinal mucosal microbiota in IBS-D with Spleen-Kidney Yang Deficiency, we utilized PICRUSt2 (Phylogenetic Investigation of Communities by Reconstruction of Unobserved States 2) software for phylogenetic analysis. The intestinal microbiome can be categorized into four main functional categories, with the median count of 39 subcategories exceeding 366.7435. Among the top 39 analyzed KEGG pathways, metabolic functions accounted for 72% of the genes (Figure 8A), primarily affecting overall and summary processes, carbohydrate metabolism, energy metabolism, nucleotide metabolism, and amino acid metabolism (Figure 8C). Although no significant changes were observed, the metabolic functions in model group were increased compared with those in normal group (Figure 8B).

### 3.9 Correlation analysis between intestinal mucosal bacteria and mucosal barrier function in IBS-D mice with Spleen-Kidney Yang Deficiency

The RDA (redundancy analysis) results indicated that *Lactobacillus* and *Lentilactobacillus* were positively correlated with D-LA, while *Clostridium*, *Pediococcus*, *Porphyromonas*, *Heminiphilus*, *Parabacteroides*, *Ligilactobacillus*, *Adlercreutzia*, and *Maribacter* showed negative correlations with D-LA. *Clostridium*, *Pediococcus*, and *Parabacteroides* were positively correlated with DAO, whereas *Pediococcus*, *Porphyromonas*, *Heminiphilus*, *Ligilactobacillus*, *Adlercreutzia*, and *Maribacter* exhibited negative correlations with DAO (Figure 8D). These findings suggest that *Lactobacillus* and *Lentilactobacillu* are correlated with intestinal mucosal barrier damage in mice following *Folium senna-*adenine administration combined with restraint-clamping tail.

## 4 Discussion

### 4.1 The combined intervention of *Folium senna*-adenine administration and restraint-clamping tail modeling induces behavioral alterations and compromises intestinal mucosal barrier integrity in mice

Observation of TCM syndrome-related behavioral characteristics in animal models serves as a critical foundation for experimental research in Traditional Chinese Medicine (Ren et al., 2020). By recording and evaluating behavioral changes of the mice during the modeling process, it was observed that model group exhibited lethargy, huddling behavior, piloerection, reduced anal temperature, damp bedding, and perianal fecal staining. From day 1 of modeling, the average water intake of model group was significantly greater than that of normal group. Starting from day 7 of modeling, the average daily food intake of model group remained consistently lower than that of normal group. Compared with pre-modeling (day 1), the body weight of normal group mice increased after modeling (day 14), while that of model group mice decreased post-modeling (day 14).

Combined with the pathological sections of the small intestinal tissues, we observed that in normal group of mice, the crypt structure was clear, and many goblet cells were present. In model group of mice, the submucosal layer and lamina propria of the intestinal tissue were separated, the muscle layer was edematous, and the arrangement of villi was disordered or absent. The number of goblet cells producing mucus in the small intestine was significantly reduced, and the mucus layer was noticeably thinner or absent. D-LA is a fermentation product of intestine-resident bacteria such as lactobacilli, whereas DAO is a highly active intracellular enzyme primarily found in intestinal mucosal villous cells. When intestinal mucosal function is impaired, both D-LA and DAO can enter the bloodstream and systemic circulation. D-LA and DAO can serve as important indicators for assessing the impairment of intestinal barrier function, and their levels are positively correlated with the degree of pathological changes in intestinal mucosal tissue (Liu et al., 2019). In this study, the serum levels of D-LA and DAO activity in model group of mice were significantly increased. Therefore, gavage of *Folium senna-*adenine administration combined with restraint-clamping tail caused changes in mouse behavior and, to some extent, affected intestinal mucosal barrier function, confirming the successful establishment of a diarrhea animal model characterized by IBS-D with Spleen-Kidney Yang Deficiency.

### 4.2 The combined intervention of *Folium senna-*adeninate administration and restraint-clamping tail stress modeling induces alterations in the intestinal mucosal microbiota composition of mice

As the most critical and complex component of the human microbial ecosystem, the intestinal microbiota plays an indispensable role in maintaining intestinal mucosal barrier homeostasis (Rea et al., 2017). Consistent with this paradigm, our experimental results demonstrated a marked reduction in mucosal-associated OTU counts in model group compared to normal group. Alpha diversity analysis revealed that the richness (Chao1 index and observed species index) and diversity (Simpson index and Shannon index) of the intestinal mucosal microbiota in model group were greater than those in normal group. Beta diversity analysis also confirmed that modeling led to changes in the overall structure of the intestinal mucosal microbial community in mice.

These results demonstrate that the modeling procedure significantly altered the intestinal mucosal microbiota characteristics, including species richness, α-diversity, and β-diversity. In terms of the composition of the dominant bacteria in the intestinal mucosa, we observed significant reductions in the relative abundances of *Bacteroidota*, *Actinomycetota*, and *Clostridium* in model mice compared to controls. These findings indicate that substantial remodeling of the microbial community structure at both phylum and genus levels follows modeling.

### 4.3 Correlations were identified between dominant bacterial genera, D-LA, and DAOc levels

LEfSe revealed differences in abundance between normal group and model group, with 11 key differentiating factors identified in normal group and 2 in model group. At the genus level, *Streptococcus*, *Helicobacter* and *Phocaeicola* were characteristic genera of normal group. The ROC curve results indicated that *Streptococcus*, *Serratia*, *Helicobacter*, *Phocaeicola*, and *Desulfomicrobium* may serve as potential biomarkers at the genus level of the intestinal mucosal microbiota and could be used as diagnostic indicators for the Spleen-Kidney Yang Deficiency syndrome in IBS-D patients. The RDA results revealed a positive correlation between *Lactobacillus*, *Lentilactobacillu*, and D-LA, as well as a positive correlation between *Clostridium*, *Pediococcus*, *Parabacteroides*, and DAO. These findings indicate that *Lactobacillus* and *Lentilactobacillu* are associated with mucosal barrier impairment following *Folium senna-*adenine administration combined with restraint-clamping tail. Therefore, we hypothesize that *Folium senna-*adenine administration combined with restraint-clamping tail disrupts the intestinal mucosal barrier (evidenced by elevated D-LA and DAO levels) and induces alterations in *Lactobacillus* and *Lentilactobacillus* populations. Therefore, their dysbiosis may serve as a key mechanism underlying mucosal damage. Based on these findings, we propose that the clinical diagnosis of IBS-D with Spleen-Kidney Yang Deficiency should integrate TCM syndrome differentiation with objective diagnostic indicators, including D-LA and DAO levels and the abundance of specific bacterial genera. For treatment, Spleen-Kidney tonifying TCM formulations may restore barrier function by modulating these microbiota, while supplementation with specific probiotics could synergistically improve therapeutic outcomes. Future studies should focus on validating these therapeutic targets in clinical cohorts to advance personalized treatment strategies for IBS-D patients with Spleen-Kidney Yang Deficiency.

## 5 Conclusion

**Figure 9:**
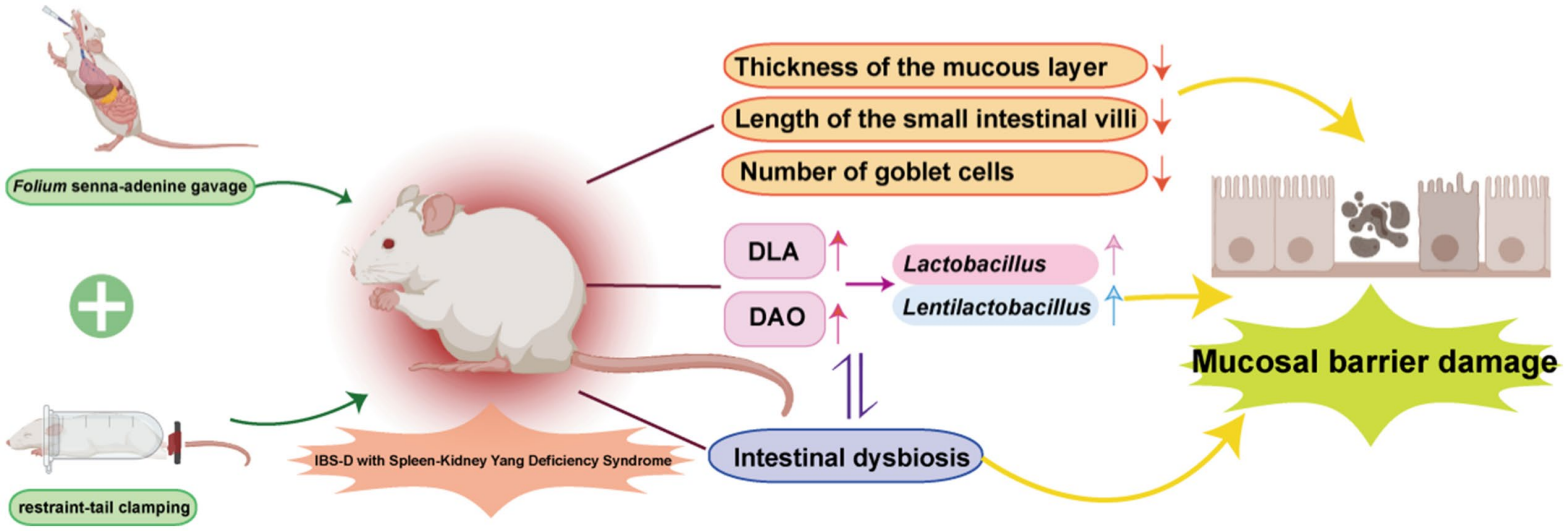
The mechanism of IBS-D with Spleen-Kidney Yang Deficiency. Adobe Illustrator 2022 was used to create the figure.

Gavage with *Folium senna-*adenine administration combined with restraint-clamping tail resulted in behavioral changes in mice, significantly damaging the structure of the intestinal mucosal barrier. This damage may disrupt the intestinal mucosal microbiota, leading to alterations in *Lactobacillus* and *Lentilactobacillus*, thereby affecting the function of the intestinal mucosal barrier (Figure 9).

## Data Availability

The original contributions presented in the study are publicly available. This data can be found here: https://www.ncbi.nlm.nih.gov/, accession number PRJNA1178577.
