# Correction: Association of intestinal mucosal barrier function with intestinal microbiota in Spleen-Kidney Yang Deficiency IBS-D mice

**DOI:** 10.3389/fmicb.2026.1877375

**Published:** 2026-06-11

**Authors:** Liwen Li, Qi Long, Na Deng, Zhoujin Tan

**Affiliations:** 1 School of Traditional Chinese Medicine, Hunan University of Chinese Medicine, Changsha, China; 2 Laboratory of Chinese Medicine Prescription and Syndromes Translational Medicine, Changsha, China

**Keywords:** Spleen-Kidney Yang Deficiency, IBS-D, intestinal mucosal barrier, intestinal mucosal microbiota, *Folium senna*

In the published article, there was an error in **Materials and methods**, Section 2.4 “*Animal grouping and model preparation*” paragraph 1. The dosage of adenine was incorrectly stated as “75 mg/kg/day.” The original sentence read as follows, “Starting from day 1 of modeling, the mice in model group received daily oral gavage of ice adenine suspension (75 mg/kg/day) for 14 consecutive days, supplemented with *Folium senna* decoction (10 g/kg/day) from day 10 to 14 (once daily) (Li et al., 2022a).” The corrected sentence should read as. “Starting from day 1 of modeling, the mice in model group received daily oral gavage of ice adenine suspension (50 mg/kg/day) for 14 consecutive days, supplemented with *Folium senna* decoction (10 g/kg/day) from day 10 to 14 (once daily) (Li et al., 2022a).”

The original version of this article has been updated.

